# P174: A model for infection control collaboration among municipalities and 3 university hospitals in the capital region, Denmark

**DOI:** 10.1186/2047-2994-2-S1-P174

**Published:** 2013-06-20

**Authors:** A-M Thye, D Mogensen, A-M Mikkelsen

**Affiliations:** 1 Clinical Microbiology, Herlev Hospital, Copenhagen, Denmark; 2 Clinical Microbiology, Herlev Hospital, Gilleleje, Denmark; 3 Clinical Microbiology, Hvidovre Hospital, Copenhagen, Denmark

## Introduction

Initiatives involved in implementing a health-care strategy that commits University Hospitals to provide general Infection Control (IC) guidance to the healthcare sector in the municipalities.

The objective was to establish 3 networks with participants from 29 municipalities, representing both the elderly and the children, IC teams from the 3 hospitals, and senior managers in hospitals and municipalities.

## Methods

Identify and contact by telephone, email or visits relevant personnel from municipalities, hospitals and relevant institutions, arrange meetings and write proposals for protocols, agendas etc. Assist the municipalities in development of IC organizations, establish a link and dialogue between the municipalities and the IC teams and arrange short educational courses to health care personal.

In order to prevent healthcare acquired infections, the network meetings and the educational courses should support IC and prevention of intersectional spread of infections and multidrug-resistant organisms, patient safety, occupational health and safety.

## Results

After a year

- 3 networks were established and municipalities are represented by 2 network participants

- 4 annual network meetings were arranged and will be customary

- 3 educational courses arranged for annually, 100 participants a year.

- Evaluation 93/100 (93%) were generally satisfied with the contents. The courses were massively oversubscribed.

A questionnaire completed by senior management in the municipalities with 21/29 responses:

- Has the initiative supported competency development? Yes: 18/21(86%), don’t know: 3/21(14%).

- Has new knowledge been disseminated in the organizations? Yes: 19/21(91%), No: 1/21(5%), don’t know: 1/21(5%).

- Is dissemination of knowledge formalized in the organization? Yes: 12/21(57%), No: 6/21(29%), Don’t know: 3/21(14%)

## Conclusion

The networks support the development of IC skills in the municipalities. Local networks are trying to disseminate new knowledge, but formalizing it, is a major challenge. There is a need for training of frontline staff. It is of great importance that need for IC counseling in the future is determined.

## Disclosure of interest

None declared.

